# Balancing Energy Budget in a Central-Place Forager: Which Habitat to Select in a Heterogeneous Environment?

**DOI:** 10.1371/journal.pone.0102162

**Published:** 2014-07-16

**Authors:** Martin Patenaude-Monette, Marc Bélisle, Jean-François Giroux

**Affiliations:** 1 Groupe de recherche en écologie comportementale et animale, Département des sciences biologiques, Université du Québec à Montréal, Montréal, Québec, Canada; 2 Département de biologie, Université de Sherbrooke, Sherbrooke, Québec, Canada; Clemson University, United States of America

## Abstract

Foraging animals are influenced by the distribution of food resources and predation risk that both vary in space and time. These constraints likely shape trade-offs involving time, energy, nutrition, and predator avoidance leading to a sequence of locations visited by individuals. According to the marginal-value theorem (MVT), a central-place forager must either increase load size or energy content when foraging farther from their central place. Although such a decision rule has the potential to shape movement and habitat selection patterns, few studies have addressed the mechanisms underlying habitat use at the landscape scale. Our objective was therefore to determine how Ring-billed gulls (*Larus delawarensis)* select their foraging habitats while nesting in a colony located in a heterogeneous landscape. Based on locations obtained by fine-scale GPS tracking, we used resource selection functions (RSFs) and residence time analyses to identify habitats selected by gulls for foraging during the incubation and brood rearing periods. We then combined this information to gull survey data, feeding rates, stomach contents, and calorimetric analyses to assess potential trade-offs. Throughout the breeding season, gulls selected landfills and transhipment sites that provided higher mean energy intake than agricultural lands or riparian habitats. They used landfills located farther from the colony where no deterrence program had been implemented but avoided those located closer where deterrence measures took place. On the other hand, gulls selected intensively cultured lands located relatively close to the colony during incubation. The number of gulls was then greater in fields covered by bare soil and peaked during soil preparation and seed sowing, which greatly increase food availability. Breeding Ring-billed gulls thus select habitats according to both their foraging profitability and distance from their nest while accounting for predation risk. This supports the predictions of the MVT for central-place foraging over large spatial scales.

## Introduction

Animals face time and energy constraints leading to trade-offs in their activity budget, which can also be modulated by factors such as the spatio-temporal distribution of food resources, conspecifics, predation risk, and phenology. How animals respond to these constraints in order to maximize their fitness through foraging behaviour has been the main focus of optimal foraging theory [1], [2]. For instance, the marginal-value theorem (MVT) has been used to predict which resource patch an animal should exploit and how long it should stay before moving to another patch or return to its nest or shelter [3], [4]. Assuming that animals maximize their net energy gain, this model has provided relevant qualitative predictions [5]. However, it has been developed and used for small-scale systems in which animals are assumed to incur few or no travel costs and to be highly informed about their environment [1], [2].

This model may therefore be difficult to apply at the landscape level because of information uncertainty about the environment, which influences learning ability and because of the limited motion and navigation capacity of animals [6], [7], [8]. For example, classical central-place foraging models based on the MVT predict that prey load size should increase with the distance traveled by a forager from its central place [4], [9]. However, a forager moving across the landscape with a large load can incur increased travel costs due to greater energy expenditures or can encounter higher predation risks through increased exposure and reduced manoeuvrability [5]. Therefore, the impact of carrying a heavy load can influence the time and energy budget of a central-place forager in different ways, sometime far from the conclusions of the classical models [10].

The MVT predicts that a foraging path is the outcome of balancing trade-offs between energy expenditures and gains, especially within landscapes where resources are heterogeneously distributed. Although it is difficult to use the MVT to make precise predictions under relaxed assumptions, classical central-place foraging models nevertheless allow to predict that distant patches must provide higher energy “prey” than those found in nearby patches [9]. Hence, the profitability of a given load size may vary for a generalist forager traveling through a heterogeneous landscape. Also, habitats providing low energy food should only be used close to the central place whereas habitats with high-energy food may be exploited near or far from the central place. It remains that travel costs may increase the use of poor quality habitats when individuals must sample and learn the quality of their environment [11], [12]. Moreover, temporal variation in habitat availability and forager condition may alter the pattern of habitat use along a distance gradient [13].

Although assessing the costs and benefits of large spatio-temporal scale movements is difficult, analytical methods based on accurate location data (e.g., GPS) are now available to study movement behaviour. Combining these analytical methods with *in situ* observations of individual foraging strategies, patch quality, and environmental conditions while considering the individuals’ characteristics has the capacity to provide insights into the cost-benefit trade-offs associated with foraging movements underlying habitat selection [13], [14]. For instance, resource selection functions (RSF) have been widely used to assess habitat selection. They are based on the comparison of relative habitat use (defined by presence-only data) and availability or on the presence/absence of individuals in habitat patches [15]. RSF are particularly informative if a distinction can be made between actively selected locations, such as foraging patches, and the incidentally selected locations visited during inter-patch movements [16], [17]. Bastille-Rousseau *et al.* [18] have advocated the use of a combination of RSF, residence time analysis, and ground surveys to study resource selection and foraging strategies at the landscape level. Considering the hierarchical aspect of the selection process, the difficulty of defining available habitats with presence-only data can be avoided by building RSF based on the habitats actually visited for foraging vs. those crossed when moving to a patch [19], [20]. Measuring the time spent by an animal within the surroundings of recorded locations (residence time) should allow discriminating between locations occurring within foraging patches and those found along movement paths [16], [21].

We used RSF and residence time analyses from GPS-tracking data, as well as survey data, diet characterization and calorimetric analyses to study the processes that determine habitat use by breeding Ring-billed gulls (*Larus delawarensis*). This species is a colonial central-place forager that feeds opportunistically upon a wide variety of prey items found in both aquatic and terrestrial habitats [22], [23]. We expected that gulls should be more likely to forage in a patch where the amount of habitats providing high-energy food increases and that such a relationship should be more pronounced far from the colony so that gulls reach a threshold of profitability. We also hypothesized that gulls should select habitats with a temporally variable food availability only when those habitats provide high food returns. For instance, agricultural lands and lawns should be selected on rainy days when annelids (earthworms) are more available to gulls [24]. By testing these predictions, our study sheds light on the process of habitat selection by animals from an energy trade-off perspective.

## Materials and Methods

### Ethics statement

Field methods to capture, mark, and collect Ring-billed gulls were approved by the Institutional Animal Protection Committee of the Université du Québec à Montréal (No.**646). The capture and marking of gulls was conducted under Environment Canada scientific permit to capture and band migratory birds (No.**10546) while the collection of specimens was carried out under Environment Canada scientific research permit (No. SC-23)

### Study area

**Figure 1:**
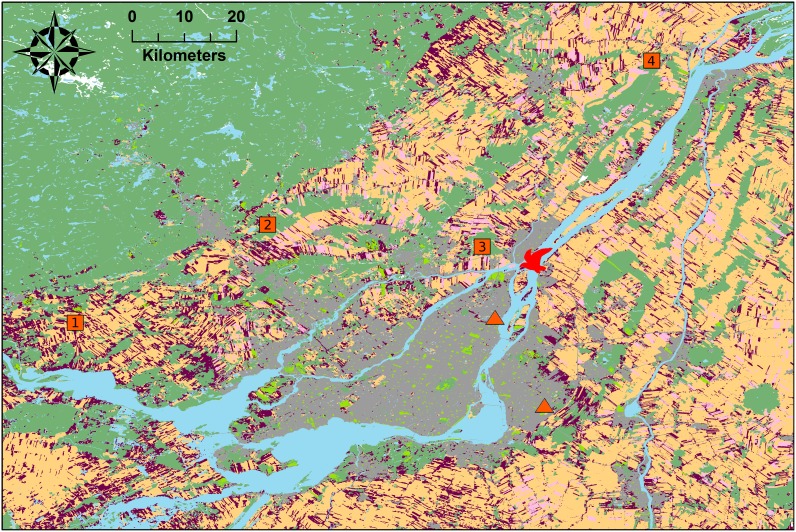
Map of the study area. Land cover types include water (blue), urban areas (gray), intensive cultures (mango), extensive cultures (purple), unidentified cultures (rose), lawns (olive green), and woodlots (dark green). Numbers in squares indicate landfill locations (1- Lachute, 2- Ste-Sophie, 3- Terrebonne, 4- St-Thomas), red triangles indicate transhipment site locations, and the bird pictogram indicates the location of the Deslauriers Island Ring-billed gull colony.

We tracked the movements of Ring-billed gulls breeding on Deslauriers Island located in the St. Lawrence River 3 km downstream from Montreal, QC, Canada (45.717°N, 73.433°W). This colony covered 11.4 ha and supported 48,000 pairs at the time of the study. The surrounding foraging area encompassed approximately 6,000 km^2^ and consisted of a mosaic of high and low density urban areas, agricultural lands of intensive (soybean, maize, and small cereals) and extensive cultures (hayfields and pastures), as well as riparian habitats along the River and its tributaries (Fig. 1). Four landfills and two waste material transhipment sites were located in the vicinity of the colony. Landfills attract gulls because of the anthropogenic food they supply but the implementation of deterrence programs may reduce their accessibility [25], [26]. During our study, the St-Thomas (41 km) and Lachute (63 km) landfills, as well as the two transhipment sites (12 and 27 km), had no deterrence program. On the other hand, the Ste-Sophie landfill (37 km) initiated a deterrence program in 2009 that combined pyrotechnics and selective culling. However, the program was limited to weekdays from 07∶00 to 15∶00 thereby leaving some feeding opportunities for gulls [26]. Lastly, the Terrebonne landfill (8 km) conducted a deterrence program since 1995 that included falconry, distress calls, and pyrotechnics. This program was in operation every day from sunrise to sunset, preventing all but few gulls to use the landfill (Thiériot, E., unpublished data).

### Telemetry

Breeding Ring-billed gulls were fitted with 10–16-g GiPSy-2 data loggers (TechnoSmart, Italy) between April and June 2009–2010. The loggers represented (mean ± SD) 2.8±0.5% of the birds’ body mass (485±49 g). Most gulls were captured and recaptured with nest traps or dip nets but some had to be recaptured by rifle shooting; carcasses were then kept for further analyses (see *Diet and calorimetric analyses*). Data loggers were attached on the two median rectrices with white TESA tape (no. 4651) and programmed to acquire locations at 4-min intervals. Tracking lasted 1 to 3 days depending on battery life. Half of the birds included in the analyses returned to their nest within 15 min after being released and 81% of them returned within 60 min. Birds that spent more than 1 h away from their nest took on average 4.5±3.9 h to return. Breeding stage upon capture was categorized as incubating or brood rearing. Gulls were sexed with genomic DNA isolation from chest feathers [27].

We recaptured 109 Ring-billed gulls (41 females, 68 males) with loggers that provided reliable data (Table S1). After removing locations within a 300-m buffer zone around the colony (see *Data analyses*), there were only 28 missing locations on a potential of 15,948. The remaining 15,920 locations had a low dilution of precision metric (DOP ≤6) and an estimated precision of ±5 m [28]. A total of 67 gulls were followed during incubation (164 foraging trips) and 42 during the brood rearing period (239 foraging trips).

### Gull Surveys

We conducted weekly surveys from April to June alternating between three periods (05∶00–10∶00, 10∶00–15∶00, and 15∶00–20∶00) to determine the proportion of time Ring-billed gulls spent foraging in their main feeding habitats. In agricultural habitats, we surveyed a 50-km roadside transect on each shore of the St. Lawrence River (*N* = 13 and 21 surveys in 2009 and 2010, respectively). We tallied the number of birds in each flock and performed an instantaneous scan sampling to determine the proportion of birds foraging (head down below the horizontal or probing into the soil) that we considered as the proportion of time spent foraging [29]. A flock was defined as a group of gulls using the same field type and not separated by more than 200 m from each other. Birds using different field types but closer than 200 m from each other were considered as different flocks. The total number of tractors and their activity (ploughing, harrowing or sowing) was also noted over the entire transect during each survey.

Observations in other habitats were conducted weekly in 2010 at fixed points located in urban (*N* = 25 points), suburban (*N* = 53 points), and riparian (*N* = 10 points) areas on the Montreal Island (*N* = 16 surveys) and along the North (*N* = 18 surveys) and the South shores (*N* = 22 surveys) of the St. Lawrence River. These sites were selected because they were susceptible to be visited by gulls while insuring that observers driving vehicles could stop safely. At each point, gulls using different habitat types (lawns, shores, water, grounds covered with concrete, asphalt or gravel, building roofs, and post lights) were counted and scanned to determine the proportion of birds foraging (erratic flight in emergent insect clouds above waterbodies, feeding on garbage, head down below the horizontal or probing into the soil or water).

Finally, we estimated the proportion of time gulls spent foraging at landfills by conducting 5-h observation periods once a week in 2009 (*N* = 7) and five days a week in 2010 (*N* = 59) at the Ste-Sophie landfill, again alternating among the three daily periods including periods with and without deterrence. Total bird counts and instantaneous scan sampling were conducted every half hour. The mean daily abundance of gulls was computed for each day as well as the proportion of birds that were actually foraging (flying less than 5 m above the active tipping area, head down below the horizontal or probing into refuse).

### Diet and calorimetric analyses

We collected 496 boli from chicks of both sexes during weekly visits to the Deslauriers colony during the rearing period of 2009 and 2010. We selected chicks haphazardly and slightly pressed their proventriculus to make them regurgitate recently swallowed food. Spontaneous regurgitations of adults (*N* = 13) captured during banding operations throughout the breeding period were also collected. Samples were frozen until they were analysed. We also kept frozen the carcasses (*N* = 51) of adults fitted with data loggers and recaptured by shooting until the content of their oesophagus and proventriculus could be analysed. Similarly, we analysed stomach contents of birds collected by rifle shooting in agricultural lands (*N* = 69), riparian areas (*N* = 54), and at the Ste-Sophie landfill (*N* = 85). We made sure that birds were actively feeding in these habitats before collecting them. For safety reasons, gulls could not be collected in urban areas. Each food item of a bolus or stomach was separated, identified, dried to constant weight and weighted (±0.01 g). Food items were grouped into broad categories (e.g., arthropods, annelids, vertebrates, refuse, vegetation, other).

Food availability could not be assessed throughout the 6,000 km^2^ of the foraging area to estimate the benefits obtained by gulls when feeding in different habitats. Instead, we relied on the relative area of each habitat and the food quality in these habitats based on energy content of the various food items. We therefore performed duplicate or triplicate calorimetric analyses of each food category using a bomb calorimeter (Parr, model 1108P).

### Data analyses

We first created a 300-m buffer zone around Deslauriers Island (colony) to discriminate between foraging trips and short movements to the shore or surrounding shallow water where gulls rest and preen [30]. Our analyses were limited to locations outside this zone. The mean number of foraging trips per day, the mean direct (Euclidean) distance between the colony and the farthest location reached during a foraging trip (whether a stopover or not), the mean distance traveled on a foraging trip and the mean sinuosity of movement paths (traveled distance divided by round trip direct distance, [31]) were compared between breeding stages and sexes using linear mixed models with gull ID as a random factor.

For each foraging trip, we calculated the total amount of time spent at different locations on the landscape by estimating residence time without rediscretization [16]. Residence time was defined as the time spent in a circle of radius *r* centred on a given location along the foraging path. The circle, with its specific habitat composition and features, could then be viewed as a potential foraging patch. In the absence of precise information regarding the spatio-temporal distribution of resources, the hierarchy of spatial scales at which animals are likely to respond to landscape heterogeneity (i.e., patches, [32]) can only be identified through behaviour [16], [33]. For each trip, we thus computed the coefficient of variation (CV) of residence times for radii ranging between 200 and 2,000 m with 100-m increments. We averaged the CV across paths and plotted them against the circle radii (Fig. S1). The mean CVs of residence time across paths showed a plateau for radii of 200 to 400 m instead of a clear peak. There was no significant difference in CVs distribution between males and females. We thus chose a 200-m radius to get a stronger contrast in habitat composition between foraging and movement patches. We finally retained locations distanced by at least two radii to limit spatial autocorrelation.

We calculated the landscape composition within each circle in which residence time was estimated based on a land cover map created in ArcGIS 9.3.1 [34] using both agricultural and topographic data ([35], [36], [37]; planimetric precision <30 m). Landscape composition was defined as the proportion of different habitats including lawns, woodlots, urban areas, and water as well as intensive, extensive and unidentified cultures. Because of their relatively small size, landfills and transhipment centres were noted as presence/absence in each circle. We also measured the distance between each location where a residence time was computed and the nest of the tracked gull. Finally, we calculated the mean daily rainfall using data from 10 meteorological stations located throughout the entire foraging area [38].

We first described the habitats within the global home range of gulls breeding on Deslauriers Island by estimating the proportion of habitats within the 100% minimum convex polygon drawn using the foraging trip locations of all birds. Next, we built a RSF based on patches visited by gulls on their foraging trips. Considering that a foraging individual must reduce its flying speed and increase its turning rate, we used residence time to discriminate “foraging patches” from “movement patches”. We assumed that if a gull spent more than 100 s in a 200-m radius circle, it was actively foraging. Otherwise, we considered that it was moving either between the colony and a foraging patch or between two foraging patches. Gulls observed foraging during surveys typically spent more than 100 s within 200 m from where they were first detected. Moreover, based on the flight speed of Black-headed gulls (*Chroicocephalus ridibundus*) and Lesser Black-backed gulls (*Larus fuscus*), which are respectively slightly smaller and larger than Ring-billed gulls (14.7–15.5 m/s, respectively; [39]), at least 26 s is required for a gull to cross a circle of 200-m radius. The remaining 74 s appears insufficient for a gull to forage significantly in such a circular patch. Although our tracking device did not allow to determine the precise activity of the birds while not moving, we consider justified to assume that gulls were actually foraging in patches where they spend more than 100 s. Indeed, during the breeding period, gulls must brood their eggs or feed their young and must therefore spend as much time as possible on the colony allowing the rest of their time to foraging.

We used mixed effects logistic regressions to quantify the influence of landscape composition on the probability that a gull foraged in a patch along its movement path. Gull ID and foraging trip ID (nested within gull ID) were treated as random factors. The addition of these terms dealt with the hierarchical structure of the data and allowed the estimation of the variability across individuals and foraging trips. Eight different models were built and compared based on the second-order Akaike information criterion (AIC*_c_*, [40]). We included the proportion of each habitat type and the occurrence of landfills and transhipment sites in all eight models. We considered the interaction of rainfall with lawns as well as with each type of agricultural cover because annelids are more prevalent under wet conditions [24]. We also included the distance between the location of a gull while foraging and its nest as a proxy for foraging costs and accessibility [41]. We considered distance both as a main effect and in interaction with the relative amount of each habitat type (except woodlots) as well as with the occurrence of landfills or transhipment sites. We used this approach because we do not know which fitness currency gulls may be maximizing and because the profitability of the different habitats may not scale linearly with distance. Although woodlots are accessible, gulls avoid being under canopy and should thus avoid forest habitats whatever the distance from the colony. We included breeding stage in interactions with each habitat type to take into consideration the gulls’ breeding phenology and their associated requirements as well as habitat phenology, particularly for agricultural cover types where farming practices and field conditions vary throughout the season. Finally, we built a second set of eight models, adding the sex of the birds in interaction with each habitat type and the distance of the patch from the colony as males and females differ in size (affecting travel costs and dominance on food patches) and provide different levels of parental care [23]. We fitted mixed effects logistic regressions using the Laplace approximation using the lme4 package (version 0.999375-39; [42]) run in the R statistical environment (version 2.12.2; [43]). AIC_c_ were computed based on maximum log-likelihoods. Multi-model inference was performed following Burnham and Anderson [41] after testing that there was no problem of collinearity.

The effect of distance on habitat selection may be non-linear partly because central-place foragers often avoid habitats near their central-place by moving further away [44], [45]. Moreover, the relative abundance of different habitats varied with distance from the colony. To overcome this problem, we first draw 1-km wide circular bands up to 67 km from the colony, which corresponds to a few kilometers further than the farthest gull location (see *Results*). For each band with at least five foraging patches, we calculated the mean area covered by each habitat type within patches to estimate habitat use. We also calculated the mean area covered by the different habitats in movement patches (≥5 patches) within each circular band. We then plotted these two values for each band and each habitat to explore habitat selection as a function of distance.

The proportion of gulls observed foraging in a flock was considered as the time spent foraging in a given habitat [29]. This proportion was modeled as a two-column matrix, with the first column giving the number of gulls foraging and the second column giving the number of gulls involved in other activities for each flock, using a GLM with a binomial error distribution and logit link function (i.e., a logistic regression) using the stats package run in R. We also assessed whether the abundance of gulls in agricultural lands was related to the total number of tractors encountered along transects, which was considered an index of agricultural field work. This was done using a GLM with a Poisson error distribution and log link function (i.e., a Poisson regression) in R. This model included tractor number, transect location (South or North shore) and their interaction as explanatory variables.

Finally, we computed the proportion of boli containing at least one item of each food category for both chicks and adults. We then calculated the mean relative amount of each food item category when present in a bolus based on dry mass. The energy value of boli (kJ) was calculated for each gull collected at the Ste-Sophie landfill, in agricultural lands and at riparian sites by combining the dry mass of each item found in the stomach and their energy value. We compared the mean energy value of boli across habitats using an ANOVA.

## Results

### Characteristics of foraging trips

The mean number of foraging trips per day was greater during the rearing period (3.1±1.0 trips/day, ±SD) than during incubation (1.9±0.8 trips/day; *t*_107_ = −7.01, *P*<0.001). The mean direct distance between the colony and the furthest location reached during a foraging trip (whether a stopover or not) was also greater during brood rearing (16.6±12.4 km, maximum = 63.5 km vs. 12.5±9.9 km, maximum = 42.4 km; *t*_107_ = −2.22, *P* = 0.03). Furthermore, the mean foraging distance traveled was greater during the rearing period compared to incubation (38.6±29.0 km, maximum = 156 km vs. 30.2±23.8 km, maximum 105 km; *t*_107_ = −2.55, *P* = 0.01). However, there was no difference in path sinuosity during the two periods (incubation: 1.2±0.2, maximum = 2.5; rearing: 1.2±0.2, maximum = 2.9; *t*_107_ = 1.38, *P* = 0.17). Finally, the mean trip duration was similar throughout the breeding period (incubation: 2.5±2.0 h, maximum = 9.6 h; rearing: 2.3±1.7 h, maximum = 12.4 h; *t*_107_ = 0.91, *P* = 0.36) but the trips lasted longer when a landfill was visited (3.5±1.8 h vs. 2.2±1.8 h; *t*_107_ = 5.95, *P*<0.0001). No significant effect of sex was found for the trip characteristics (all *P*>0.21).

### Habitat selection

The composition of foraging and movement patches was highly variable (Table 1). Nevertheless, both movement and foraging patches were on average composed of smaller percentages of woodlots and of intensive and extensive cultures than what was found over the whole foraging range. An opposite trend was found for urban areas, waterbodies, landfills, and transhipment sites. While woodlots and urban areas covered a smaller proportion of foraging patches than movement patches, intensive cultures were relatively more important in foraging patches. Landfills and transhipment sites also occurred more often in foraging than movement patches. The distribution of residence time was strongly skewed to the right, with a peak under 100 s and a maximum reaching 19,377 s or 5.4 h (Fig. S2).

**Table 1:** Cover percentage of eight habitat types available in the foraging range of 109 Ring-billed gulls breeding on Deslauriers Island established as the minimum convex polygon calculated with all gull locations and mean cover percentage (±1 SD) in movement (residence time <100 s) and foraging (residence time ≥100 s) patches (200-m radius), 2009–2010.

	% cover		
	Foraging range	Movement patches	Foraging patches
Habitat type	(5,565 km^2^)	(*N* = 2,599)	(*N* = 4,490)
Lawns (parks, golf courses, etc.)	1.2	1.8±8.3	2.1±9.4
Woodlots	20.6	13.7±28.8	4.8±16.0
Urban areas	16.8	27.8±38.5	23.1±36.6
Water bodies	5.3	18.7±34.8	22.4±37.9
Intensive cultures	39.5	24.3±34.1	31.4±38.7
Extensive cultures	11.7	8.4±16.2	8.0±15.6
Unidentified cultures	4.1	3.4±12.3	4.2±14.3
Landfills/Transhipment sites	0.1	1.3±11.3^a^	4.2±20.0^a^

a Percent occurrence.

Model ranking based on AIC*_c_* remained similar when considering the sex of individuals and its interaction with habitat types or patch distance. Yet, we only show results for models without sex as they performed better with much less parameters (ΔAICc = 3.04). The best model (*w_i_* = 0.813) included habitat types as well as the distance separating the foraging patch from the colony, the breeding stage and their two-way interactions with habitat types (Table 2). The model that also included rainfall and its interaction with habitat types scored as the second best model (*w_i_* = 0.187), leaving barely any support from the data for the remaining models. Note that the same two models were selected with similar, strong levels of evidence for other residence time thresholds (60, 80, 120, and 140 s) and with patch radii of 200 and 400 m, underlining the robustness of our results with respect to these two assumptions.

**Table 2:** Summary of *a priori* models based on resource selection functions that predict the probability that a breeding Ring-billed gull will forage in a patch (200-m radius) for a 100-s residence time threshold.

Model	Deviance	K	ΔAICc	*w_i_*
H+D+B	7,891	26	0.00	0.813
H+D+R+B	7,886	30	2.94	0.187
H+D	7,940	19	35.06	0.000
H+D+R	7,936	23	38.63	0.000
H+B	8,466	18	558.98	0.000
H+R+B	8,459	22	560.26	0.000
H	8,533	11	611.33	0.000
H+R	8,527	15	613.67	0.000

H: habitat types; D: distance between a location and the colony; R: mean daily rainfall; B: breeding stage (egg incubation vs. chick rearing); K: number of parameters; *w_i_*: Akaike weight.

**Figure 2:**
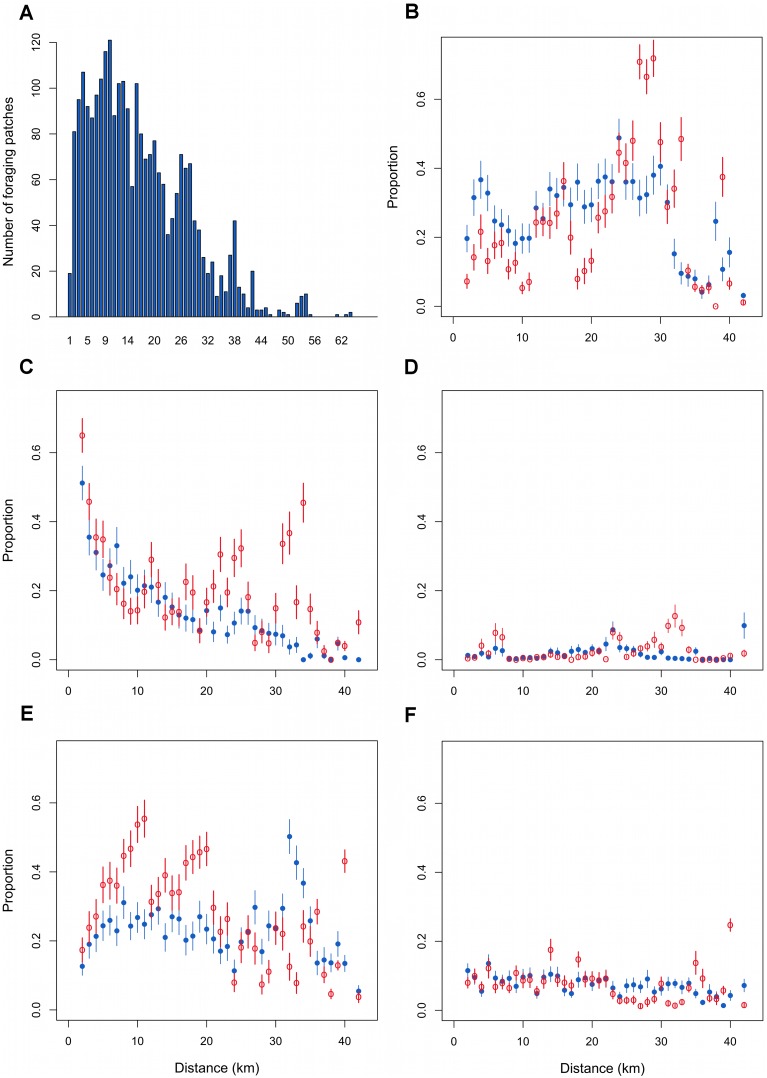
The effect of distance on habitat selection by foraging Ring-billed gulls. Number of foraging patches within 1-km concentric bands from the colony (a). Mean (±1 SD) proportion of urban areas (b), waterbodies (c), lawns (d), intensive cultures (e), and extensive cultures (f) in foraging (blue) and movement patches (red) in relation with the distance from the colony.

Ring-billed gulls had a greater probability of foraging in patches located farther from the colony (Table 3). The distribution of these patches with respect to their distance from the colony was skewed to the right and showed a noticeable mode at ∼10 km notwithstanding habitat types (Fig. 2a). Not surprisingly, gulls strongly avoided foraging in patches that included large amounts of woodlots (Table 3). Patches containing urban areas were significantly avoided close to the colony but increasingly selected further away. In fact, gulls tended to forage in patches with more urban cover compared to movement patches when the birds were between 25 and 35 km from their nest site (Fig. 2b). Because the colony was surrounded by water and gulls foraged little near the colony, there was a significant overall avoidance of this habitat (Table 3). Nevertheless, there was nearly a significant positive interaction between waterbodies and distance. In fact, waterbodies were relatively more important in foraging patches compared to movement patches when the birds were at 12 km or more from the colony (Fig. 2c). As expected, Ring-billed gulls foraged to a greater extent in patches containing lawns on rainy days (Table 3). We observed a greater proportion of lawns in foraging than in movement patches at around 5 km and again between 27 and 35 km (Fig. 2d).

**Table 3:** Mixed-effects averaged logit resource selection functions quantifying the probability that a breeding Ring-billed gull forage in a patch.

Variable	*β*	SE	95% CI	
Intercept	−0.612	0.491	−1.575	0.351
Distance*	0.089	0.013	0.063	0.115
Woodlots*	−2.963	0.448	−3.840	−2.086
Lawns	−0.212	0.834	−1.846	1.422
Urban areas*	−2.550	0.521	−3.570	−1.529
Landfills	0.992	0.510	−0.007	1.992
Water*	−1.130	0.516	−2.142	−0.118
Extensive cultures	−1.037	0.619	−2.251	0.177
Intensive cultures	−0.901	0.516	−1.913	0.111
Unidentified cultures	−0.319	0.688	−1.666	1.029
Lawns×Distance	0.017	0.033	−0.049	0.083
Urban areas×Distance*	0.067	0.016	0.036	0.098
Landfill×Distance	0.001	0.017	−0.033	0.035
Water×Distance	0.031	0.016	−0.001	0.063
Extensive cultures×Distance	−0.005	0.021	−0.045	0.035
Intensive cultures×Distance	−0.026	0.015	−0.056	0.004
Unidentified cultures×Distance	−0.036	0.026	−0.087	0.015
Lawns×Incubation	−0.050	0.671	−1.365	1.266
Urban areas×Incubation	0.210	0.244	−0.267	0.688
Landfill×Incubation	0.271	0.446	−0.603	1.145
Water×Incubation	0.205	0.254	−0.294	0.703
Extensive cultures×Incubation	−0.349	0.418	−1.168	0.469
Intensive cultures×Incubation*	1.429	0.237	0.965	1.893
Unidentified cultures×Incubation	0.911	0.474	−0.018	1.839
Lawns×Rainfall*	0.038	0.019	0.001	0.075
Extensive cultures×Rainfall	0.000	0.007	−0.013	0.013
Intensive cultures×Rainfall	−0.002	0.004	−0.009	0.005
Unidentified cultures×Rainfall	−0.003	0.008	−0.019	0.012

Model-averaged coefficients (*β*), unconditional standard errors (SE), and 95% confidence intervals (CI) are presented. Variables followed by an asterisk are significant (95% CI excluding 0).

**Figure 3:**
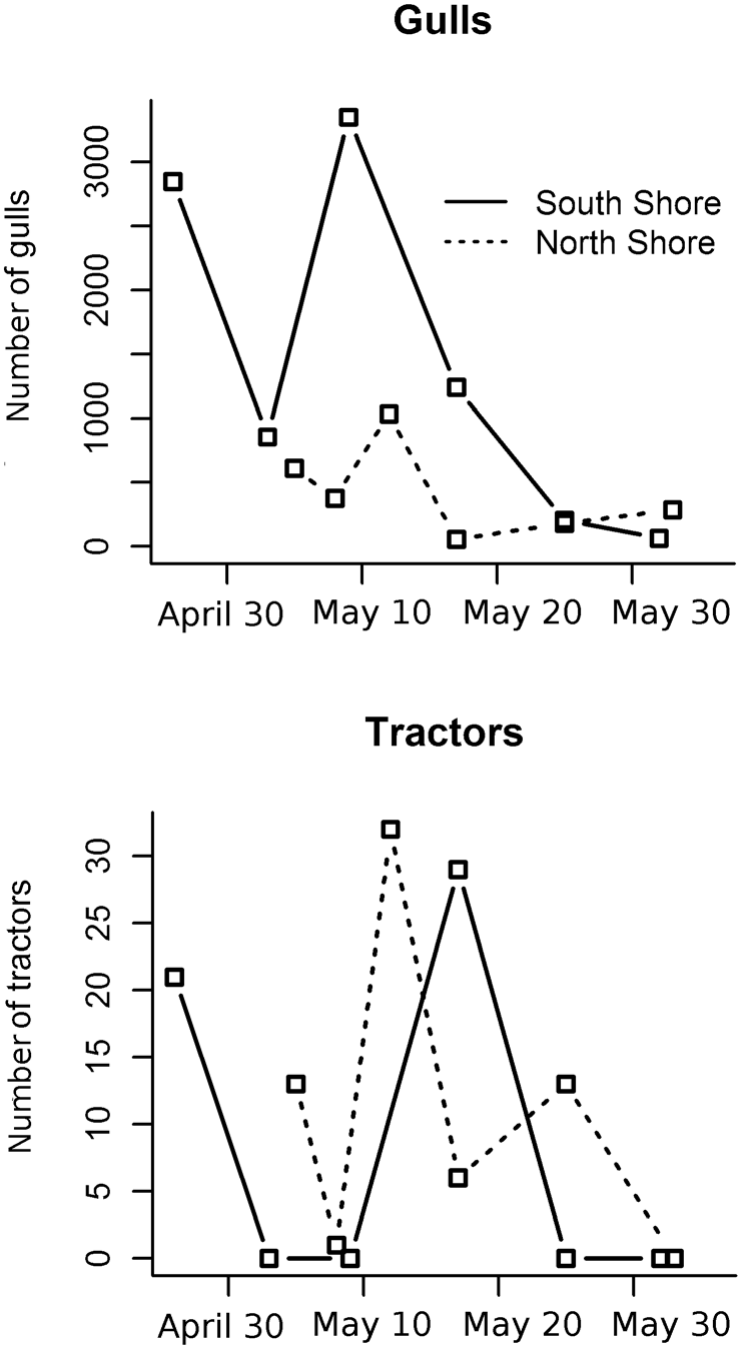
Use of agricultural lands by breeding Ring-billed gulls. Number of gulls and of tractors observed during surveys on the North and South shores of the St. Lawrence River, 2010.

**Figure 4:**
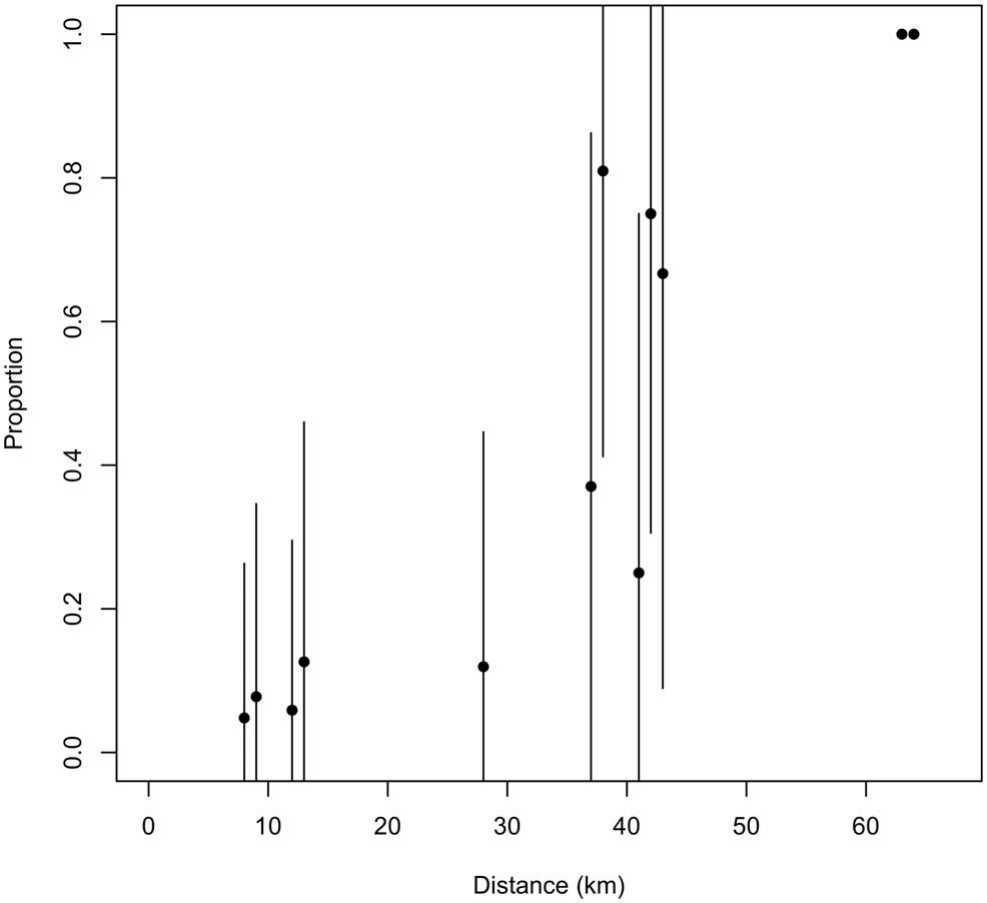
Use of landfills and transhipment sites by Ring-billed gulls. Mean (±1 SD) proportion of Ring-billed gull locations at landfills and transhipment sites in foraging patches within 1-km concentric bands located at different distances from the colony. All landfills and open transhipment sites were visited by at least one tagged individual. Some sites encompassed more than one band.

The probability that a gull foraged in a patch increased with the proportion of intensive cultures (i.e., cereal fields) during incubation but tended to decrease during chick rearing (Table 3). More specifically, there were more intensive cultures in foraging than in movement patches up to 23 km from the colony (Fig. 2e). Similarly, the likelihood that gulls foraged in patches with extensive cultures (i.e., hayfields and pastures) tended to decrease with increasing amounts of this habitat (Table 3). The effect of distance on the use of extensive cultures by foraging gulls was not important (Fig. 2f). Gull surveys conducted in agricultural landscapes support the above patterns as the presence of gulls in intensive agricultural lands was related to the occurrence of ploughing, harrowing, and sowing, which all took place during the incubation period (mid-April to mid-May; Fig. 3). Indeed, the number of gulls observed along transects in agricultural lands increased with cultivation activities as indexed by the number of operating tractors seen in the fields (Poisson regression: *β ±* SE = 0.064±0.001, *z* = 53.9, *P*<0.01). Of 20,900 gulls counted along transects, 52% were observed on bare soil fields (ploughed or recently sown), 34% on cereal fields with short vegetation (<10 cm), 8% on stubble cereal fields and the remaining 6% on recently mowed hayfields. Finally, Ring-billed gulls had a greater tendency to forage in patches where a landfill or transhipment site was present (Table 3) and this was especially true as distance from the colony increased (Fig. 4).

### Foraging behaviour, diet, and energy

The mean proportion of time that Ring-billed gulls spent foraging varied among habitats (deviance = −1.5×10^4^; df = 1351, *P*<0.01). It was higher in agricultural lands (0.54±0.40) than in landfills and transhipment sites (0.17±0.20; *z* = −76.9, *P*<0.01), riparian habitats (0.12±0.25; z = −78.4, *P*<0.01), urban areas (0.15±0.32; *z* = −53.4, *P*<0.01) and on lawns (0.43±0.41; *z* = −3.1, *P*<0.01).

**Figure 5:**
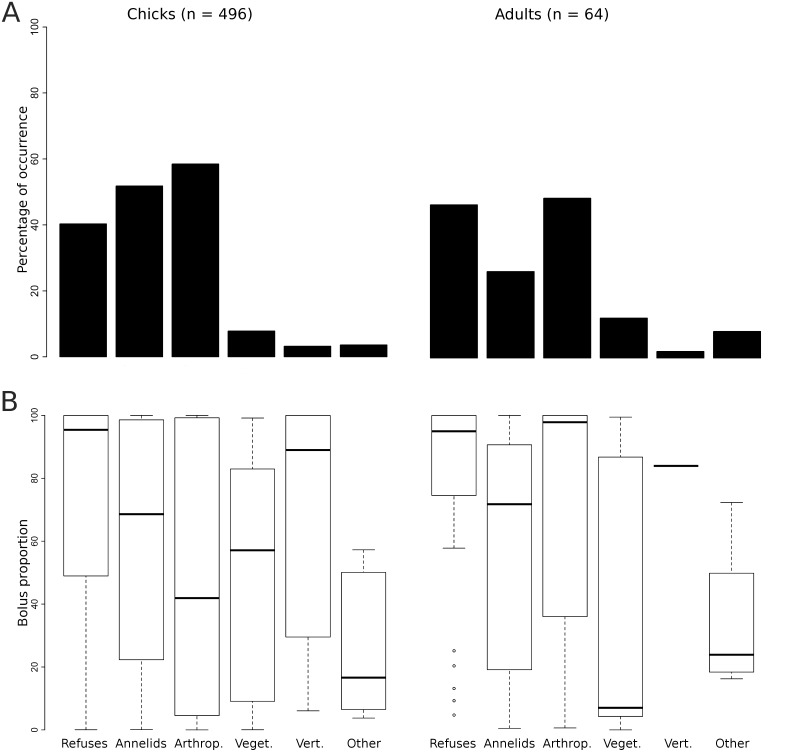
Diet of chicks and breeding adults of Ring-billed gulls. Diet of Ring-billed gull chicks (boli) and breeding adults (boli and stomach contents) at the Deslauriers Island colony, 2009–2010, expressed as the percentage of occurrence of each food category (a) and the proportion based on dry mass of each food category when present (b). Boxplots provide the first (bottom line), second (black midline) and third (top line) quartiles; whiskers extend to observations found up to 1.5 times the interquartile range; observations outside this range are indicated by empty dots.

The four main food items (i.e., refuse, annelids, arthropods, and vegetation) were found in 40–60% of the boli collected from chicks reared on Deslauriers Island (Fig. 5a). The same items were found in the stomachs and boli of breeding adults, but in lower proportions (25–30%); it was compensated by a greater frequency of vertebrates and miscellaneous items. Yet, vertebrates occurred in less than 10% of the boli/stomachs in both chicks and adults. When refuse items were present, they contributed to a large proportion of the contents based on dry mass, unlike vegetation and miscellaneous items that usually represented a small proportion (Fig. 5b). The importance of annelids and arthropods was much more variable when present. Vertebrates were also quite variable in chick boli, whereas they clearly contributed to a very large proportion of the adult diet when they occurred.

Stomach contents from gulls collected in landfills were largely composed of fat meat typically found in refuse (Table 4). In agricultural lands, stomach contents were composed more or less equally of annelids and grains (soybean and corn). Stomach contents from riparian areas contained edible refuse, wild fishes, and arthropods. By pooling data on the relative importance of each food item and their respective energy content, we found that the mean energy value of stomach contents differed significantly among habitats (*F*_2,144_ = 3.51, *P* = 0.03). It was significantly higher in landfills (112.8±169.8 kJ) than in agricultural lands (55.8±63.6 kJ) and riparian areas (56.5±97.5 kJ), which were not significantly different.

**Table 4:** Mean dry mass (g ± SD) and mean energy value (kJ) of nine food items gathered by sub-adult and adult Ring-billed Gulls in three habitat types (*N* = number of birds with food items present in their stomach).

	Mean dry mass (g)			
Food items	Landfills	Agricultural lands	Riparian habitats	Energy
	(*N* = 81)	(*N* = 54)	(*N* = 22)	(kJ)
Meat	1.84±4.71	-	0.87±2.81	30.1
Bread/rice	1.27±2.62	-	0.47±1.05	19.9
Potatoes/French fries	0.15±0.79	-	0.32±0.89	21.8
Miscellaneous refuse	0.79±2.69	-	-	22.6
Annelids	0.10±0.32	1.99±2.80	-	13.8
Arthropods	0.06±0.24	0.03±0.08	0.09±0.24	22.8
Corn/soybean grains	-	1.44±3.36	-	18.1
Vertebrates	0.01±0.11	-	0.46±0.61	19.4
Miscellaneous	0.10±0.32	0.08±0.35	-	21.3
TOTAL	4.47±6.37	3.55±3.68	2.36±3.39	-

## Discussion

By combining analyses of GPS-tracking data and information on the gulls’ abundance, diet, and proportion of time spent foraging in different habitats, we found that the distance from the colony and habitat phenology had strong effects on the process of habitat selection by breeding Ring-billed gulls foraging in a heterogeneous environment. For instance, they positively selected areas managed intensively for agriculture at a distance up to about 23 km from the colony but only when fields were being ploughed, harrowed, or sown. Gulls also selected areas where landfills and transhipment sites were present, especially as the distance from the colony increased. The mean energy intake being significantly greater in landfills than in agricultural lands, these results clearly suggest a trade-off by Ring-billed gulls to balance their energy budget. The St. Lawrence River and its tributaries are often used as passageways when flying to and from the insular colony, which resulted in a general avoidance of this habitat as feeding site. Over 12 km, however, gulls may stop along the shores of the rivers and the lakes or feed on emergent insects over water resulting in a selection of this habitat.

### Energy trade-offs in selected habitats

The spatial and temporal variation in food availability could not be measured across the 6,000-km^2^ study area. Nevertheless, we believe that using energy as an index of food quality and the relative area covered by each habitat allowed us to assess the relative benefits of different habitats. The strong selection for intensive cultures during incubation corresponded to the period when fields were being cultivated and the new cereal shoots were still at a height that allowed the birds to feed without visual obstruction. This seems to be associated with the occurrence of short periods of high food availability. Although it is difficult to differentiate the confounding effects of the breeding stage from the timing of field work and food availability, the positive effect of soil preparation and seed sowing on the abundance of gulls in agricultural lands during the incubation period (vs. brood-rearing) supports the hypothesis that selection for a specific habitat is higher during the peak of food availability. During our surveys, most gulls foraged in bare soil fields as observed for Black-headed gulls [46]. Moreover, half of the gulls’ diet in agricultural lands was made of annelids, which are more accessible when tractors are ploughing and harrowing. Sibly and McCleery [24] have shown a positive relationship between the abundance of Herring gulls (*Larus argentatus*) in agricultural lands and the biomass of earthworms near the ground surface. Yet, the averaged RSFs did not detect an effect of rainfall on the use of agricultural lands despite the positive effect of ground wetness on the availability of annelids and their use by gulls [24]. In agricultural fields, gulls rely on the presence of heavy machinery that cannot work on wet soils. This contrasts with the use of lawns by gulls that was strongly associated with rainfall. Although we could not sample birds using urban areas, the greater availability of annelids on rainy days on lawns and their use by gulls is well established [22]. The other half of the gulls’ diet in agricultural lands was made of soybeans and corn, which availability increases when sowing takes place (e.g., seeds accidentally dropped along road and field edges when farmers fill their seeders and seeds sown in superficial ground; M. Patenaude-Monette, pers. obs.). Annelids, soybeans and corn composed a less energy-rich diet than the food gathered by gulls at landfills. Considering that gulls selected the intensive agricultural lands no further than 23 km, we suggest that the profitability of this habitat was limited by the travel costs associated with the distance from the colony and the relatively low energy value of the food.

Gulls selected areas comprising landfills or transhipment sites throughout the breeding season, a period during which food availability at these sites does not vary with time. Although the accessibility (distance from the colony and deterrence program effectiveness) and volume of refuse differed among sites, we could only account for variation in distance from the colony. The selection of landfills was stronger, but also more variable, as the distance increased. Thus, the selection of landfills was probably not constrained by their distance from the colony as was the selection of agricultural lands at the scale of the study area. Nevertheless, its high variability suggests that not all gulls used landfills and transhipment sites. Indeed, landfills and transhipment sites were present in less than 5% of foraging patches of all individuals. Moreover, when refuse food items occurred in boli, they accounted for a much larger proportion of the bolus than any other food items. Furthermore, both the mean bolus mass and the mean energy content of food were much higher in landfills than in any other habitats.

We can hypothesize that gulls incur higher travel costs when foraging in landfills, which are located farther from the colony than agricultural lands [47]. Habitat accessibility is indeed likely to be negatively correlated with the distance separating the foraging site from the nest as travel costs (time, energy) increase with distance [41], [48]. Accordingly, intensively managed agricultural lands may thus provide a profitable net energy gain to foraging gulls despite food items of lower energy value, at least during the incubation period. On the other hand, landfills with their more energy rich food may be valuable foraging sites despite their remoteness and are thereby selected by gulls. The stronger selection observed with increasing distance to the colony (up to 63 km) may result from the fact that the closest sites (<30 km from the colony) included two transhipment sites where refuse is less available than at landfills. Moreover, the Terrebonne landfill that received the largest tonnage of refuse and which is located the closest to the colony has a very effective deterrence program (É. Thiériot, unpublished data).

### Time constraints in urban areas

Gulls are known to feed on refuse in commercial and residential areas and on handouts offered by citizens [49]. Nevertheless, we found that breeding Ring-billed Gulls avoided foraging in urban areas located <10 km from the colony, but showed the opposite trend at greater distances. This pattern may result from the profitability of urban areas as foraging sites, which likely depends on the type of development (e.g., residential, commercial, or industrial) and population density. The proportion of time foraging was indeed very low in urban areas where gulls adopted a sit-and-wait strategy to exploit spatially and temporally scattered feeding opportunities (e.g., people handouts and overfilled garbage bins). While the proportion of time foraging was comparable in urban areas and in landfills, foraging opportunities are probably much less predictable in the former habitat. Furthermore, commercial and residential areas of high population densities (i.e., with greater foraging opportunities) were located about 20 km from the colony, which is much further than the closest landfill or agricultural lands. Although urban refuse food may present high energy contents, the time to gather enough refuse is likely too long to make foraging trips to urban areas profitable, particularly during the rearing period when chicks are waiting to be fed at the colony [50], [51]. The situation may nevertheless be different during the post-breeding period when gulls are then actively using urban areas ([52], C. Girault and J.-F. Giroux, unpublished data).

## Conclusion

Combining RSF to survey data, diet characterization, and calorimetric analyses allowed us to characterize habitat selection processes of a central-place forager from an energy trade-off perspective. It also shows that other factors such as predation risk associated to deterrence programs at landfills can also play a role in the process of habitat selection at large spatial scales as suggested through the concept of landscape of fear [53]. This approach was applied to a species that had to move over a large area to find food in a heterogeneous environment where habitat profitability also varied in time. Despite the complexity brought up by travel costs and habitat sampling issues, we were able to show that classical optimal foraging theory can make qualitative predictions applicable at the landscape level. This adds to the few evidences that optimal foraging theory has the potential to be scaled-up to the landscape level as predicted by Lima and Zollner [6]. Moreover, once classical models will have been modified such that their constraints are adapted to large spatio-temporal scales (e.g., [11], [54], [55]), GPS data loggers will allow us to test these models by linking the foraging behaviour of individuals to their breeding performance [14]. Such progress would make significant strides toward understanding the links between movement behaviour, habitat selection, fitness, and population dynamics within heterogeneous landscapes. For instance, this approach could be applied to many gull populations around the world to link their dynamics to food availability through landfill, agriculture, and fishery management.

## Supporting Information

Figure S1 Residence times of breeding Ring-billed gulls in relation with patch size. Mean coefficient of variation (CV) of residence times within circular patches of different radii centred on locations obtained by GPS data loggers (*N* = 109 birds).
(TIF)

Figure S2 Frequency distribution of residence times of breeding Ring-billed gulls. Residence times were established for 200-m radius circular patches centred on locations obtained by GPS data loggers (*N* = 109 birds).
(TIF)

Table S1 Characteristics of individual Ring-billed gulls tracked during the study.
(PDF)
