# Supplementary figures and images for: Balancing Energy Budget in a Central-Place Forager: Which Habitat to Select in a Heterogeneous Environment?
Source: PLoS One. 2014 Jul 16;9(7):e102162. doi: 10.1371/journal.pone.0102162 (PMC4100874; doi:10.1371/journal.pone.0102162)

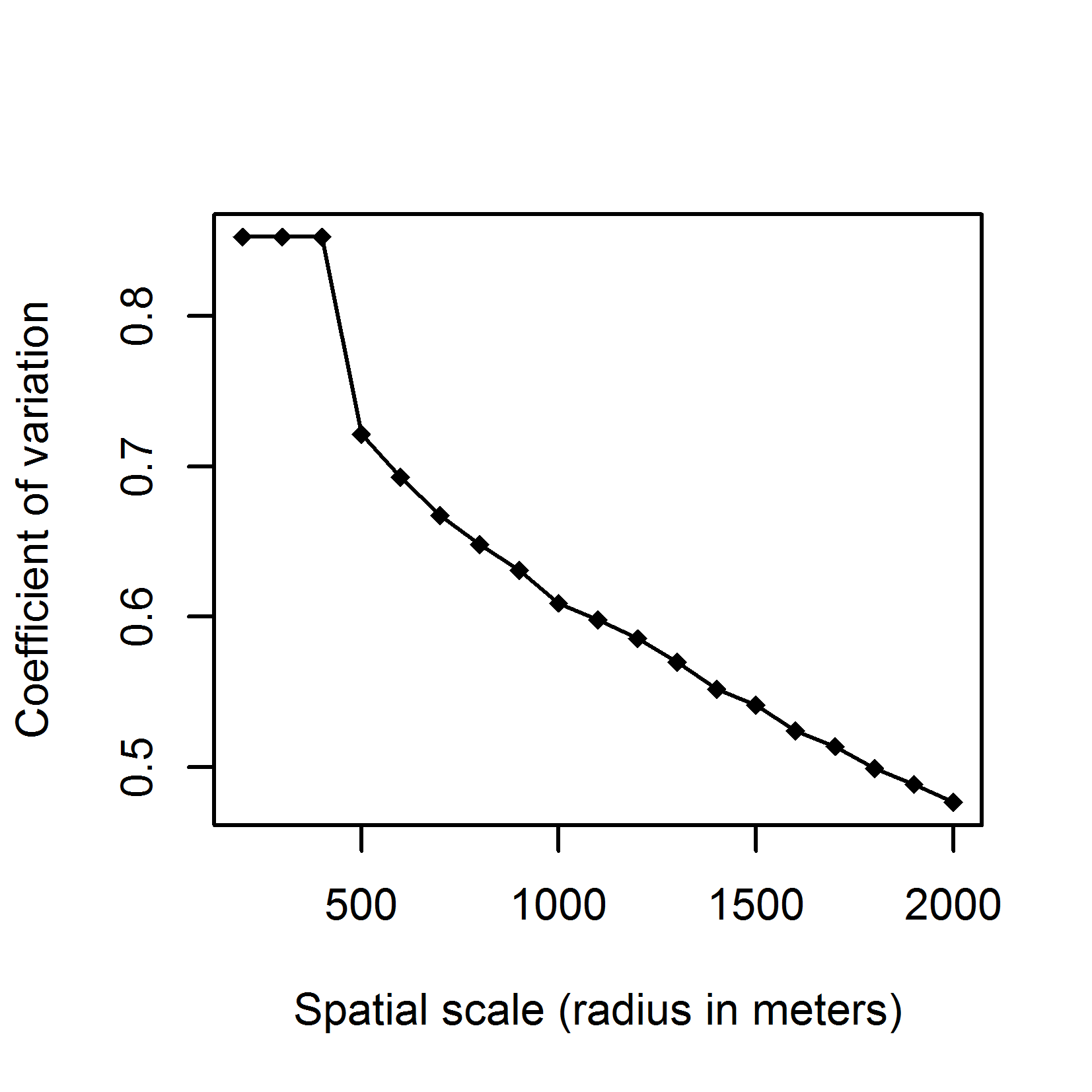

Supplement: Figure S1 — Residence times of breeding Ring-billed gulls in relation with patch size. Mean coefficient of variation (CV) of residence times within circular patches of different radii centred on locations obtained by GPS data loggers (N = 109 birds). (TIF) [file pone.0102162.s001.tif]
