# Supplementary material for: Balancing Energy Budget in a Central-Place Forager: Which Habitat to Select in a Heterogeneous Environment?
Source: PLoS One. 2014 Jul 16;9(7):e102162. doi: 10.1371/journal.pone.0102162 (PMC4100874; doi:10.1371/journal.pone.0102162)
Supplement: Table S1 — Characteristics of individual Ring-billed gulls tracked during the study. (PDF) [file pone.0102162.s003.pdf]

Table S1. Characteristics of individual Ring-billed gulls tracked during the study.

Gull ID	Sex	Year	Date marking	Breeding stage	Time to return to nest (min)	Tracking duration (h)	Nb trips	Total nb locations	Nb locations used
1	Male	2009	01-Jun	Brood rearing	60	30.8	4	460	168
2	Male	2009	08-Jun	Brood rearing	648	31.0	2	467	208
3	Female	2009	08-Jun	Brood rearing	4	8.0	1	123	31
4	Female	2009	08-Jun	Incubation	196	27.3	2	410	133
5	Male	2009	12-Jun	Brood rearing	52	30.0	4	445	262
6	Male	2009	12-Jun	Incubation	52	33.0	2	497	98
7	Male	2009	15-Jun	Brood rearing	1	31.0	3	462	153
8	Female	2009	17-Jun	Brood rearing	1	33.0	4	488	225
9	Female	2009	17-Jun	Brood rearing	1	33.0	4	492	208
10	Male	2009	19-Jun	Brood rearing	4	31.0	4	455	177
11	Female	2009	19-Jun	Brood rearing	9	31.5	2	472	114
12	Male	2009	23-Jun	Brood rearing	1	32.5	5	485	238
13	Female	2010	26-Apr	Incubation	1	32.5	1	487	73
14	Male	2010	26-Apr	Incubation	851	32.5	2	485	106
15	Male	2010	26-Apr	Incubation	289	31.8	1	477	57
16	Male	2010	26-Apr	Incubation	1	30.0	2	448	45
17	Male	2010	26-Apr	Incubation	33	31.5	2	475	56
18	Female	2010	26-Apr	Incubation	88	33.8	2	507	75
19	Male	2010	28-Apr	Incubation	9	36.5	2	548	46
20	Female	2010	28-Apr	Incubation	32	31.0	1	466	116
21	Female	2010	28-Apr	Incubation	1	33.3	4	1006	240
22	Male	2010	28-Apr	Incubation	1	33.3	3	501	136
23	Male	2010	28-Apr	Incubation	1	32.0	2	483	101
24	Male	2010	28-Apr	Incubation	62	31.8	1	474	27
25	Male	2010	28-Apr	Incubation	1	26.0	1	1006	118
26	Female	2010	30-Apr	Incubation	204	32.5	2	456	140
27	Male	2010	30-Apr	Incubation	1	32.3	2	485	78
28	Male	2010	30-Apr	Incubation	25	33.0	4	493	125
29	Male	2010	30-Apr	Incubation	21	34.0	4	508	70
30	Male	2010	30-Apr	Incubation	8	31.5	4	474	152
31	Male	2010	30-Apr	Incubation	3	31.3	3	451	117
32	Female	2010	30-Apr	Incubation	15	32.5	2	490	130
33	Female	2010	01-May	Incubation	12	68.7	9	1044	226
34	Male	2010	01-May	Incubation	77	30.5	2	1021	140
35	Male	2010	01-May	Incubation	689	24.0	3	335	65
36	Male	2010	01-May	Incubation	20	33.0	3	523	37
37	Female	2010	03-May	Incubation	44	33.3	3	498	85
38	Female	2010	03-May	Incubation	1	34.0	2	700	85
39	Male	2010	03-May	Incubation	4	34.5	2	458	110
40	Male	2010	03-May	Incubation	8	32.3	1	486	74
41	Female	2010	04-May	Incubation	8	31.0	2	466	21
42	Male	2010	04-May	Incubation	4	29.0	2	434	36
43	Female	2010	04-May	Incubation	1	30.5	3	461	118
44	Male	2010	05-May	Incubation	64	31.7	1	468	38
45	Male	2010	05-May	Incubation	20	30.8	3	463	86
46	Male	2010	05-May	Incubation	12	34.5	2	515	66
47	Male	2010	05-May	Incubation	1	24.5	1	367	41

Table S1. Characteristics of individual Ring-billed gulls tracked during the study.

Gull ID	Sex	Year	Date marking	Breeding stage	Time to return to nest (min)	Tracking duration (h)	Nb trips	Total nb locations	Nb locations used
48	Male	2010	05-May	Incubation	69	29.5	2	445	116
49	Male	2010	07-May	Incubation	51	33.5	2	502	54
50	Female	2010	07-May	Incubation	52	34.3	2	517	146
51	Male	2010	07-May	Incubation	9	66.0	5	994	270
52	Female	2010	10-May	Incubation	4	34.0	2	511	138
53	Female	2010	10-May	Incubation	76	32.5	3	489	100
54	Male	2010	10-May	Incubation	4	32.8	2	490	125
55	Male	2010	10-May	Incubation	16	22.0	3	332	54
56	Male	2010	10-May	Incubation	68	43.3	3	649	156
57	Male	2010	11-May	Incubation	11	31.5	1	473	14
58	Male	2010	11-May	Incubation	1	32.0	3	478	179
59	Female	2010	11-May	Incubation	1	27.0	3	406	81
60	Male	2010	13-May	Incubation	24	31.5	3	475	44
61	Female	2010	13-May	Incubation	40	35.5	4	530	85
62	Female	2010	13-May	Incubation	384	30.3	2	456	81
63	Male	2010	13-May	Incubation	13	62.5	6	951	139
64	Male	2010	16-May	Incubation	24	65.3	4	981	142
65	Male	2010	16-May	Incubation	13	34.0	6	513	70
66	Female	2010	16-May	Incubation	24	31.0	3	468	30
67	Male	2010	18-May	Incubation	160	20.5	1	308	25
68	Male	2010	18-May	Brood rearing	1	31.0	4	494	105
69	Male	2010	18-May	Brood rearing	24	31.0	6	466	118
70	Female	2010	18-May	Brood rearing	24	33.5	5	508	121
71	Female	2010	18-May	Brood rearing	200	31.5	6	471	211
72	Female	2010	20-May	Incubation	1	36.0	3	544	148
73	Male	2010	20-May	Brood rearing	17	65.0	9	973	97
74	Male	2010	20-May	Brood rearing	20	35.0	4	522	149
75	Male	2010	20-May	Brood rearing	4	31.3	5	469	125
76	Female	2010	21-May	Incubation	1	9.3	1	141	27
77	Male	2010	23-May	Brood rearing	60	63.0	4	945	151
78	Female	2010	23-May	Brood rearing	20	68.3	9	1027	305
79	Male	2010	23-May	Brood rearing	1	67.5	10	1009	357
80	Male	2010	23-May	Brood rearing	3	54.5	6	968	206
81	Female	2010	23-May	Brood rearing	1	63.0	7	948	248
82	Male	2010	23-May	Brood rearing	1	64.8	9	972	176
83	Male	2010	23-May	Incubation	1	67.5	2	1013	132
84	Male	2010	23-May	Brood rearing	40	62.0	9	934	268
85	Male	2010	26-May	Brood rearing	280	53.8	1	800	187
86	Male	2010	26-May	Brood rearing	120	63.3	4	921	104
87	Male	2010	26-May	Brood rearing	20	68.0	11	1025	412
88	Male	2010	26-May	Brood rearing	8	31.5	5	472	136
89	Male	2010	26-May	Brood rearing	36	58.5	5	518	219
90	Male	2010	26-May	Brood rearing	24	32.3	5	480	179
91	Male	2010	28-May	Brood rearing	1	66.3	7	994	290
92	Female	2010	28-May	Incubation	4	65.0	8	977	353
93	Female	2010	28-May	Brood rearing	320	63.7	7	954	330
94	Female	2010	28-May	Incubation	8	33.0	3	493	84

Table S1. Characteristics of individual Ring-billed gulls tracked during the study.

Gull ID	Sex	Year	Date marking	Breeding stage	Time to return to nest (min)	Tracking duration (h)	Nb trips	Total nb locations	Nb locations used
95	Female	2010	28-May	Brood rearing	4	32.5	4	486	166
96	Female	2010	28-May	Brood rearing	1	32.8	4	492	175
97	Female	2010	31-May	Brood rearing	16	68.0	14	1018	370
98	Male	2010	31-May	Incubation	16	64.3	5	971	338
99	Male	2010	31-May	Brood rearing	9	68.5	10	1028	401
100	Female	2010	31-May	Brood rearing	268	32.7	5	492	141
101	Female	2010	31-May	Incubation	605	18.0	1	248	83
102	Female	2010	31-May	Brood rearing	1	32.0	7	484	181
103	Male	2010	31-May	Brood rearing	9	26.0	2	390	86
104	Male	2010	03-Jun	Incubation	12	38.8	1	581	288
105	Female	2010	03-Jun	Brood rearing	8	63.7	12	956	307
106	Female	2010	03-Jun	Incubation	21	65.0	7	974	270
107	Male	2010	03-Jun	Incubation	20	32.5	4	488	188
108	Male	2010	03-Jun	Brood rearing	53	35.5	6	531	255
109	Female	2010	03-Jun	Brood rearing	32	30.5	4	458	133
